# Ensuring universal access to quality care for persons with presumed tuberculosis reaching the private sector: lessons from Kerala

**DOI:** 10.1186/s12939-024-02151-1

**Published:** 2024-05-17

**Authors:** P. S. Rakesh, Mohd Shannawaz

**Affiliations:** 1 Amity Institute of Public Health & Hospital Administration, Amity University, Noida, India; 2 The Union South East Asia Office, New Delhi, India

**Keywords:** Public private partnership, Private sector engagement, Schedule H1, Standards of TB care, STEPS, Universal access to TB care

## Abstract

**Background:**

More than half of the people with Tuberculosis (TB) symptoms in India seek care from the private sector. People with TB getting treatment from private sector in India are considered to be at a higher risk for receiving suboptimal quality of care in terms of incorrect diagnosis and treatment, lack of treatment adherence support with a high loss to follow-up rate that could eventually increase their risk of drug resistance. The current study aims at documenting the approach and efforts taken by the Kerala state to partner with the private health care delivery providers for ensuring quality TB care to the people with presumed TB reaching them.

**Methods:**

A case study approach was adopted with review of all available literature followed by five Key Informant Interviews to understand the case through a primary descriptive exploration. Grounded theory approach was used to generating the single theory of the case itself that explains it.

**Results:**

Kerala state has taken a variety of interventions to ensure universal access to TB care for citizens reaching the private sector with documented improvement in the quality of TB care. Key learnings from these initiatives were (i) patients need to be at the centre of partnerships, (ii) good governance is essential for ensuring Universal Health Coverage in a mixed health system, (iii) data intelligence is required to guide partnerships, (iv) identification of the correct ‘problems’ is crucial for effective design of partnerships and (v) a platform for meaningful dialogue of key stakeholders is needed.

**Conclusion:**

Kerala experience demonstrated that if governments take a proactive role in engaging the private sector, in an informed and evidence-based way, they can leverage the advantages of the private sector while protecting the public health interest.

**Supplementary Information:**

The online version contains supplementary material available at 10.1186/s12939-024-02151-1.

## Background

The Standards for TB Care in India (STCI), which is a locally customized version of the International Standards of Tuberculosis Care*,* mentions 26 standards of care that every citizen of India should receive irrespective of the sector of treatment [1]. STCI deliberate on standard tools and strategies for early and complete detection of TB, standards of treatment in terms of drugs and regimens for best patient outcome, standards of public health actions and patient support such as adherence monitoring, clinical follow up, contact investigations and TB Preventive Therapy to the family. STCI lays down what is expected for quality TB care from the Indian healthcare system.

More than half of the people with TB symptoms in India seek care from the private sector [2, 3]. Gaps in the TB care cascade such as people with TB not having access to correct and complete diagnosis, people diagnosed with TB not being started on correct treatment; and people started on treatment not completing treatment, were observed more among people with TB who were diagnosed in the private sector compared to the public sector [4–7]. There are also concerns about the suboptimal quality of care including incorrect diagnosis and non-standardized treatment regimens, lack of systems for treatment adherence support resulting in high loss to follow-up rate increasing the risk of drug resistance among the people who seek care from the private sector in India [4–7]. Many services assured in the public sector, such as free diagnostics including rapid molecular tests and drug susceptibility testing, free quality assured drugs, treatment adherence support and monitoring, contact investigation and TB preventive therapy, linkages to social welfare schemes rarely reach the patients who are treated in the private sector. Not having access to full range of quality TB care when and where the people need them, could be a hindrance to achieve Universal health coverage (UHC).

Realising the urgent need to engage private sector, the National Strategic Plan (NSP) for TB Elimination in India (2017–2025) enlisted various strategies to ensure that patients reaching the private sector receive care as per the STCI [8]. National TB Elimination Program (NTEP) has taken a variety of approaches including trainings, regulations, provisions of free services such as drugs and diagnostics, incentives and partnership schemes to engage with private sector [9]. Government of India has issued directives making TB notification mandatory as a first step to ensure STCI to all people affected with TB [10]. Provisions have been developed in Ni-kshay, the real time case-based web-based management information system of NTEP, where private providers can directly log in to the system using their user credentials and notify TB and report outcomes [11]. NTEP provides 500 INR (IUSD = 85 INR) as an incentive to the private provider to notify each TB patient and another 500 INR to report the treatment outcome [12]. Anti TB medicines have been included in schedule H1 which can only be sold on prescription of a Registered Medical Practitioner and details of the prescriber, the patient and the drug sold needs to be maintained by the chemists [13]. NTEP has also provisions for supplying free quality assured anti- TB drugs and free molecular tests and drug susceptibility tests to the patients reaching the private sector. Directly and through various agencies, NTEP intends to provide support for contact investigations and treatment adherence to all patients reaching private sector. NTEP guidance document on partnership (2019) details out about the purchase of various services from private sector [14]. Patient Provider Support Agencies (PPSA), predominantly Non-Governmental Agencies, are contracted in with the task of engaging with private sector for notifying cases to NTEP and coordinating with NTEP for free drugs and follow-up.

Despite all these initiatives in India, TB notifications and ensuring public health actions for people with TB have not been really converted into routine practice in private sector. NSP intended to achieve a target of two million TB notification from private sector in 2021; there is a gap of 1.3 million TB cases who are ‘missing’ in the surveillance system [2, 9]. Studies, annual reports and evaluations revealed that Kerala state has successful ensured quality TB care to the people reaching the private sector [9, 15, 16]. The current study aims at documenting the approach and efforts taken by the Kerala TB Elimination program to partner with the ‘for profit’ formal private health care delivery providers for ensuring quality TB care with special emphasis on potential lessons to be learnt from the state. This is expected to help policy makers and programme managers in other parts of India and in other countries who face challenges in partnering with private sector for ensuring universal access to quality TB care.

## Methods

### Local setting

Kerala has experienced a 7.5% annual decline in the TB incidence since 2015 and has been certified for the same by Government of India under its sub-national TB free certification process [17, 18]. The state TB program has been appreciated by the Joint Monitoring Mission (JMM) 2019, led by the World Health Organization and global developmental partners, for providing patient-centric TB care through systematic interventions [15]. Various efforts by the state for providing support to the people and family with TB were documented [19]. The private health care sector in Kerala accounts for more than 70% of all facilities and 60% of all inpatient beds. A significant proportion of people from low socio economic status also avail health care from private hospitals in the state for various reason such as long waiting time in public facilities, perceived low quality of care in public sector and proximity of health facilities [20]. National TB Prevalence survey (NTPS) revealed that among those with TB symptoms and sought health care in Kerala, 38% consulted the private sector first [2].

### Design

A case study approach was used to understand the case through a primary descriptive exploration. Review of all available literature followed by five Key Informant Interviews (KIIs) were conducted.

### Desk review

Desk review of all available documents and studies were attempted first. All documents such as policy documents, operational plans, guidelines, annual reports and evaluation studies available in public domain in official websites of health department were referred to look for (1) policy and plan for engagement of private sector for TB care and (2) outcomes and data related to TB patients reaching the private sector. A high level of objectivity and sensitivity was maintained during the process; the authenticity of the documents were assessed, and the goals and biases were explored before examining the content. Literature search was done in MEDLINE, Embase and Web of Science using the key words (TB OR Tuberculosis) AND (Private hospital OR Private facility OR Private Partnership OR Private Practise OR Private Sector OR Public Private Partnership) AND Kerala. The search was carried out on 21st December 2022, and all studies including descriptive and analytical studies were included irrespective of the publication type. Studies published since 2000–2022 were included as our interest was in the recent situation, and earlier publications were difficult to access. Reference lists of papers identified in the searches, were scanned. Information was obtained about studies from state TB cell from where permissions for using TB-related data are sanctioned.

We used the operational definition provided by World Health Organisation which defined private health sector as ‘the individuals and organizations that are neither owned nor directly controlled by governments and are involved in provision of health services’ [21]. Reading abstracts, studies related to private sector and TB done in Kerala were included. We focussed on ‘for-profit’ healthcare providers, because they are more numerous and difficult to engage. We included only health service providers rather than manufacturers or distributors of medical equipment, technologies, consumables or drugs. All identified articles were screened for full-text review. Articles not focussing on ‘for-profit’ health sector and ‘quality of TB care’ were excluded.

### Key informant interviews

Based on the literature review, additional five KIIs were conducted to fill in gaps in the information, to explore additional documents to be reviewed and to validate the observations from the literature. Key informants were persons with long experience in the field of TB control, who had broad knowledge of the TB scenario in the state, were aware of the TB programme activities over the years and who were involved in policy formulation and implementation related to TB and private sector. They were selected as they were the persons with the longest experience, each having more than 15 years of experience, in the field and had a broad overview of the TB and private sector engagement in the state. They were (i) a senior bureaucrat, involved in policy making (ii) a state-level programme manager responsible for TB programme activity implementation, (iii) a technical consultant for TB elimination from a global partner agency, responsible for guiding the state in TB policy formulation, implementation and supervision (iv) a senior leader of Indian Medical Association who was associated with TB projects implemented through IMA and (v) director of a 600 bedded private hospital. Participants were approached by phone, the purpose of the interviews were communicated and the prerequisites during the interview (stable internet connection, calm environment and with video switched on) were communicated. Two interviews were conducted in English and the rest in Malayalam, by PS (male, medical doctor with post-graduation in public health with 10 years of research experience), who was experienced in conducting qualitative studies and was fluent in the local language. All KIIs were conducted online using Zoom (Zoom Video Communications, San Jose, CA, USA) with video. One researcher recorded the proceedings, identifying key themes and monitored the verbal and non-verbal interactions. All participants who were contacted could participate. Major themes discussed were initiatives taken by the Government to engage private sector, major outcomes of such strategies and reasons for changing strategies. Each interview lasted for approximately 45 min (range: 25–75). No repeat interviews were attempted. KIIs were later transcribed verbatim and translated into English. Transcripts were returned to participants for comments.

Grounded theory approach was used to develop theory. We did not attempt to generate strong theories rather focussed on generating the single theory of the case itself that explains it. This was a deliberate attempt in order not to draw away our attention from features for understanding the case.

The transcripts were then manually coded by PS, emerging themes were identified. Citations with similar coding were grouped according to the predetermined themes. Themes were derived from the data. Recurrent themes were marked as important. All the flagged statements were put together and synthesised. The team went through the transcripts and notes and reached a consensus. Data triangulation was done comparing information from literature and qualitative interviews. Inductive thematic saturation was reached and no new codes emerged after the fourth interview. The study was approved by the Institutional Ethics Committee of Amity University, Noida, India.

## Results

**Fig. 1:**
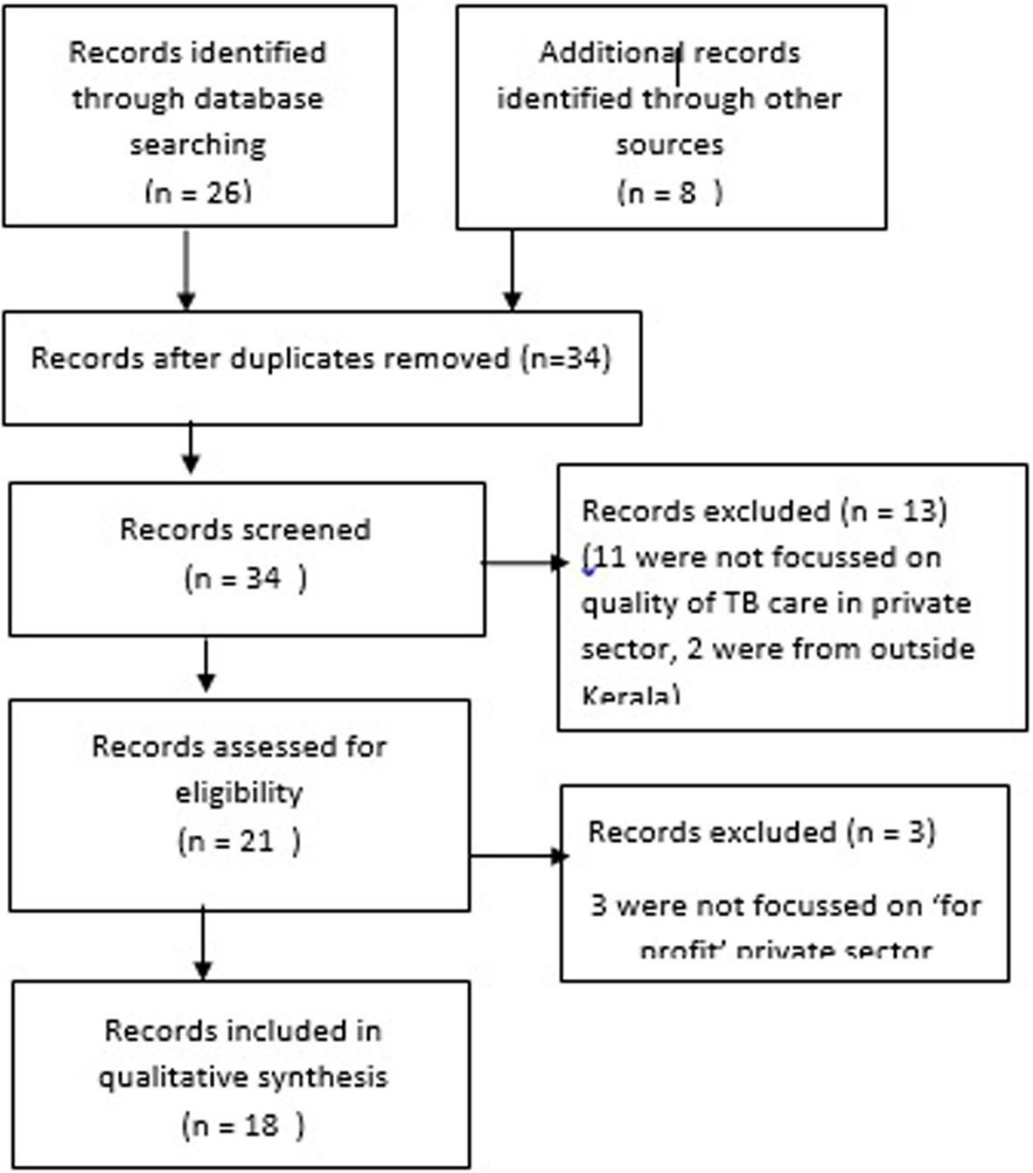
PRISMA flowchart indicating the results of literature search

PRISMA flow chart for the literature review were presented as Fig. 1.

The results of desk review and the KIIs are presented below.

### Efforts for private sector engagement for TB care in Kerala

**Fig. 2:**
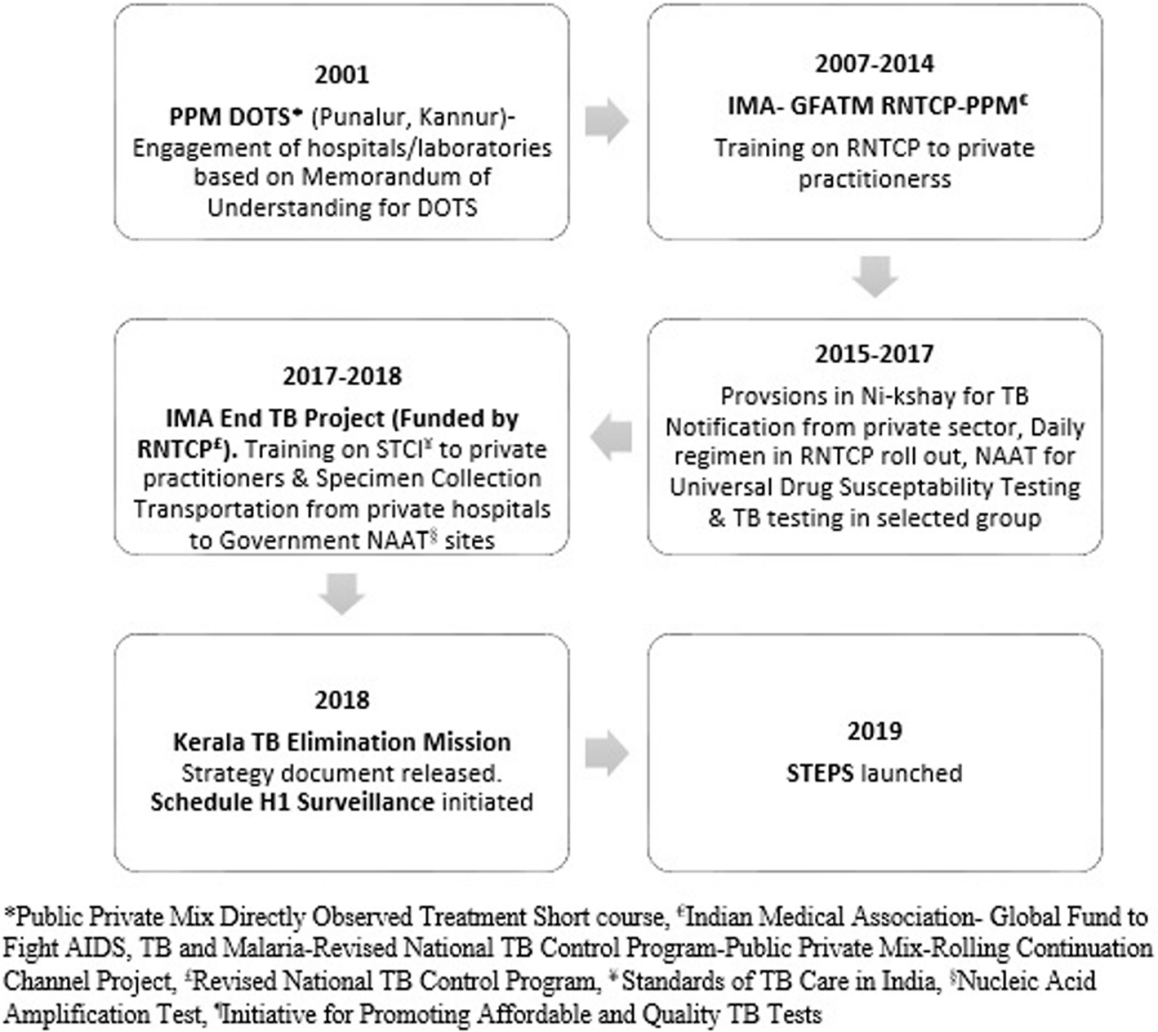
Milestone of Private Partnership for TB Care in Kerala

Many public-private mix strategies for TB care started in Kerala during the initial years of implementation of the National TB Elimination Program (NTEP), then called Revised National TB Control Program (RNTCP). Milestones of various initiatives by the state TB program to engage private sector for TB care ares described in Fig. 2. Details of major initiatives by NTEP directly or in partnership with various stakeholders are compiled in Table 1.

**Table 1:** Description of major initiatives to partner with private sector for TB care in Kerala

Name of the Initiative	Major Stakeholders	Modality of Engagement	Major Outcomes	Remarks
PPM DOTS Model* (2001–2015)	District TB Program & Private Hospitals/laboratories	TB Program provided training and support for 100 private laboratories/hospitals and encouraged referral of people with TB to public sector facilities for treatment based on a signed partnership scheme.	Increase in case detection which contributed to approximately 15% of all diagnosed TB patients in the state [22–24]	Coverage was limited to small hospitals and laboratories predominantly run by the leaders of IMA, Teaching hospitals, Missionary and NGO run hospitals [25]
IMA GFATM RNTCP PPM RCC^€^ (2007–2014 )[26]	State & District TB Program & IMA	Training of Private practitioner on RNTCP. 2000 Private Practitioners trained.	Generated goodwill for National TB Program among private sector[27]. Approximately 30% of private doctors refer diagnosed TB to RNTCP[27].	No substantial increase in TB Notification or delivery of public health actions to the patients reaching the private sector.
IMA End TB Project (2017–2018)	State and District TB Program & IMA	Training of private practitioners on STCI Specimen collection transportation system connecting private hospitals with Government NAAT sites	More than 90% of private sector doctors prescribed regimen following STCI [27]. Uptake of NAAT from private sector increased [9]	Finding the utility, the specimen collection system was later institutionalised directly by NTEP.
Schedule H1 Surveillance [28]	State & District TB Program, Drugs Controller department, Chemist’s association	Enforced a regulatory measure to document every sales of anti-TB drugs. Periodic Sensitisation and education for individual chemists through one to one and group meetings	Identification of potential providers for engagement and identification of ‘missing’ cases outside the surveillance system closing down the gap to near zero [28]	Feeling of being under surveillance further improved the quality of prescriptions outside the program.
STEPS [29, 30]	Government of Kerala, State & District TB Programs, Private Hospital Managements through Private Hospital Consortium, Professional Medical Associations through Coalition of Professional Medical Associations, Project JEET	446 hospitals initiated self-initiated business promotion and customer loyalty services to people with TB, blended with the social responsibility of the private sector [30]. NTEP provides services customised to each private hospital’s demand for the patients such as free diagnostics, free drugs, domestic air borne infection control kits, support for contact tracing, linkages to social welfare schemes, HIV counselling and testing [30]	Improvement in quality of care to people with TB reaching private sector as evidenced by improvement in (i) microbiological confirmation of cases, (ii) rifampicin resistance testing at baseline, (iii) percentage of those informed of their HIV status, (iii) percentage of those with their treatment outcome reported [9, 30, 31]	

In 2016, even after the NTEP implemented a daily anti-TB regimen—the absence of which was cited as the major reason by the private sector for not participating—there was not much improvement in the private sector participation in terms of contributions towards TB notification or partnering with NTEP on signed partnership schemes. Operations researches conducted through the NTEP mechanisms, revealed many challenges for sub-optimal private sector participation [32, 33]. Efforts were also put in place to understand the real problems of patients reaching the private sector [34]. A study that followed up a cohort of TB patients treated in private facilities in Kerala reported a loss to follow-up of 20–30% [34]. Limited ability to monitor and promote treatment adherence remained a major challenge in the private sector. Although NTEP documents the treatment outcome of every patient diagnosed/enrolled for treatment, such documentation is rare in the private sector. Lack of a network of field staff in the private sector limits the ability to monitor and support adherence to standards of care. STEPS (System for TB Elimination in Private Sector) evolved as a solution for ensuring standards of TB care in a patient-centric way for all patients accessing the private sector, addressing the concerns of the public and private sector [29, 30]. A pilot STEPS centre in a private tertiary care centre in Kerala demonstrated that establishing STEPS centre within the hospital ensured 100% TB notification with a 4-fold increase in the number of patients notified over 6 months [35].Two interventions which emerged as prominent and replicable ones in our review are described below.

### STEPS model

STEPS was envisioned as an equal partnership between the public and private sector for benefit of the society with improving standards of TB care as the outcome. Concept and evolution of STEPS are documented in detail elsewhere [30]. STEPS has three components: (i) a private hospitals TB consortium at district and state level consisting of hospital managements for policy support and review (ii) a coalition of medical professional associations at state and district levels for advocating with doctors and (iii) a STEPS centre in each private hospital. STEPS centre within a private hospital is a single window for diagnostic and treatment services, notification, patient linkage with social welfare, contact investigation, TB Preventive Therapy and treatment adherence support. A central person (STEPS lead) nominated by the hospital management, work together with contact persons (STEPS links) for each in-house department in a hub-and-spoke model. The STEPS lead and links are typically staff nurses. STEPS Links from various in-house clinical departments transfer the patients and related information to the STEPS Lead. Patients visit STEPS centre where education, counselling, support and linkages for molecular diagnostics, anti TB treatment initiation, contact investigations, TB Preventive Therapy, social welfare schemes and air borne infection control counselling are provided. STEPS Lead follows up the patient periodically over telephone and provides treatment adherence support, monitors adverse drug reactions, reminds clinical follow up and schedules reviews. Patient visits the concerned in house departments for clinical follow up. Through STEPS, private hospitals proactively supported patients to make decisions and participate in their own care. It also fosters customer loyalty. STEPS Lead enters information in NI-KSHAY. NTEP through its field staff and also through the five city officers of project JEET, a PPSA worked in the state for 2 years (2018–2019), provided customised and need based support to the patients reaching private hospitals, through the STEPS centres, including free diagnostics such as molecular tests and drug susceptibility testing, transportation of specimen from the hospitals for testing, free drugs, support for contact investigation, TB Preventive Therapy, Direct Benefit Transfer, linkages to social welfare services and retrieval if there is a lost to follow up. STEPS was implemented in all 14 districts of Kerala state, India since January 2019. Of the 446 hospitals mapped which manages more than 80% of the TB patients in the state, 318 established STEPS centres during 2019 and the remaining in 2020 [17, 30].

### Outcomes of STEPS

JMM (2019 November, India) visited Kerala and recommended supporting the establishment of STEPS Centres in all private health care facilities [15]. A formal evaluation of STEPS was conducted by a multi-disciplinary team in 2021 by (i) visiting 30 randomly selected STEPS centres for assessing infrastructure and process using a checklist, (ii) validating the patient data with Ni-kshay by telephonic interview of 57 TB patients (iii) analysing the quality of patient care indicators (iv) conducting in-depth interviews with 33 beneficiaries and stakeholders to understand their satisfaction and perceived benefits and (v) a cost analysis from the perspective of NTEP, private hospital and patients [31] .

**Fig. 3:**
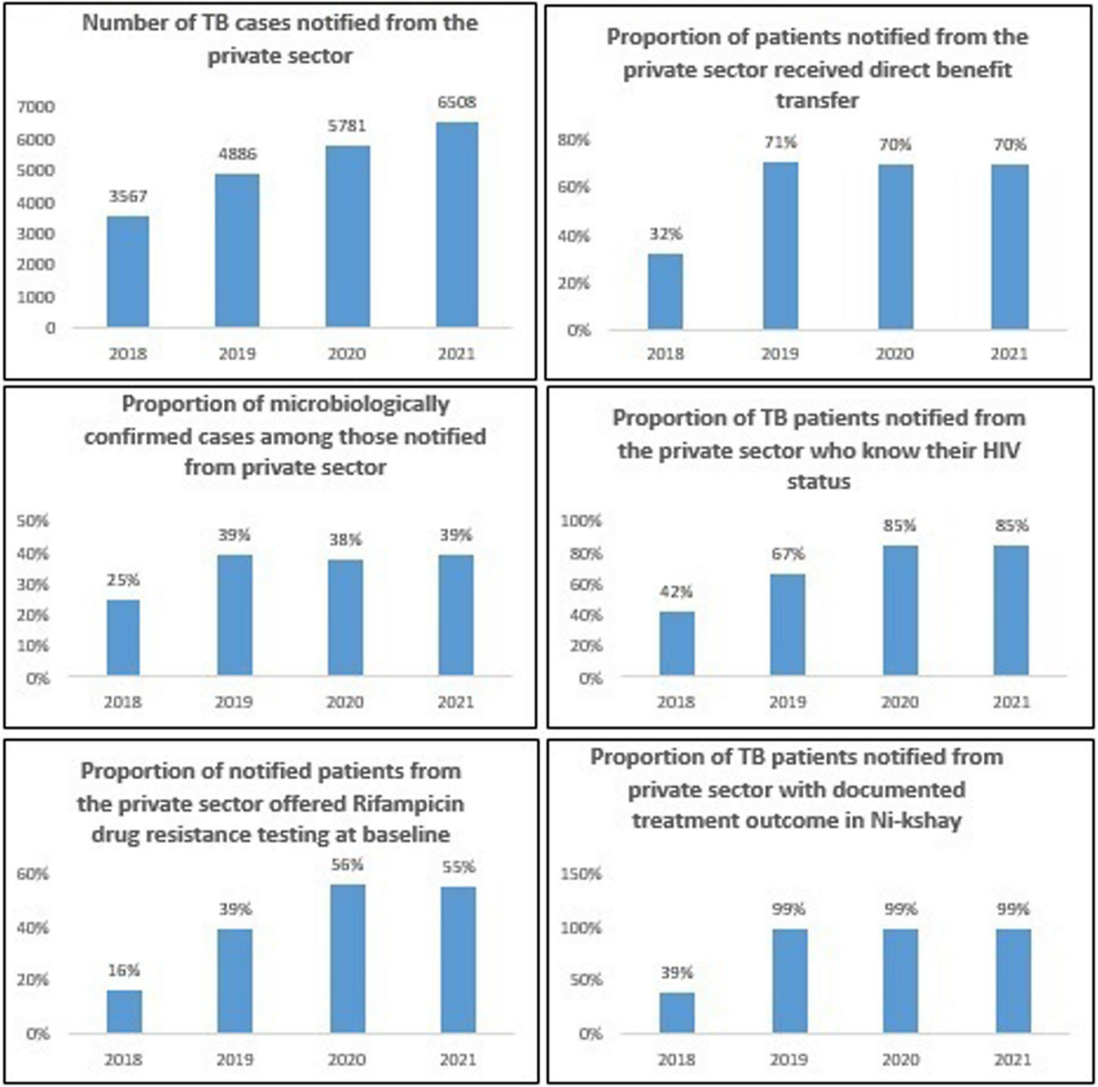
Trend in the indicator for ‘quality of care’ among the patients with TB who availed care from private sector in Kerala (2018–2021)

Evaluation concluded that STEPS is a low cost, ‘patient centric’ model where the patient can approach any provider according to his/her choice and get uniform high quality TB care. Evaluation also revealed that STEPS was acceptable to all stakeholders and patient satisfaction was good. There was significant improvement in the quality of TB care indicators for patients diagnosed in private hospitals over the years in terms of notification, proportion with a microbiological confirmation of diagnosis, known HIV status, beneficiary receiving baseline rifampicin testing, Direct Benefit Transfer and the documented treatment success rate (Fig. 3).

Number of specimens from private sector tested using molecular testing machines at public laboratories increased from 7606 in 2018 to 14,940 in 2021 [9]. STEPS led to a shift from using private anti-TB drugs to NTEP-supplied drugs and there was a drastic drop in the sale of anti-TB drugs in the state from 1.6 million rifampicin units in 2018 to 0.5 million rifampicin units in 2019 [16, 17, 31]. STEPS had also demonstrated good resilience in ensuring TB services during COVID-19 pandemic period [36].

### Schedule H1 surveillance

**Fig. 4:**
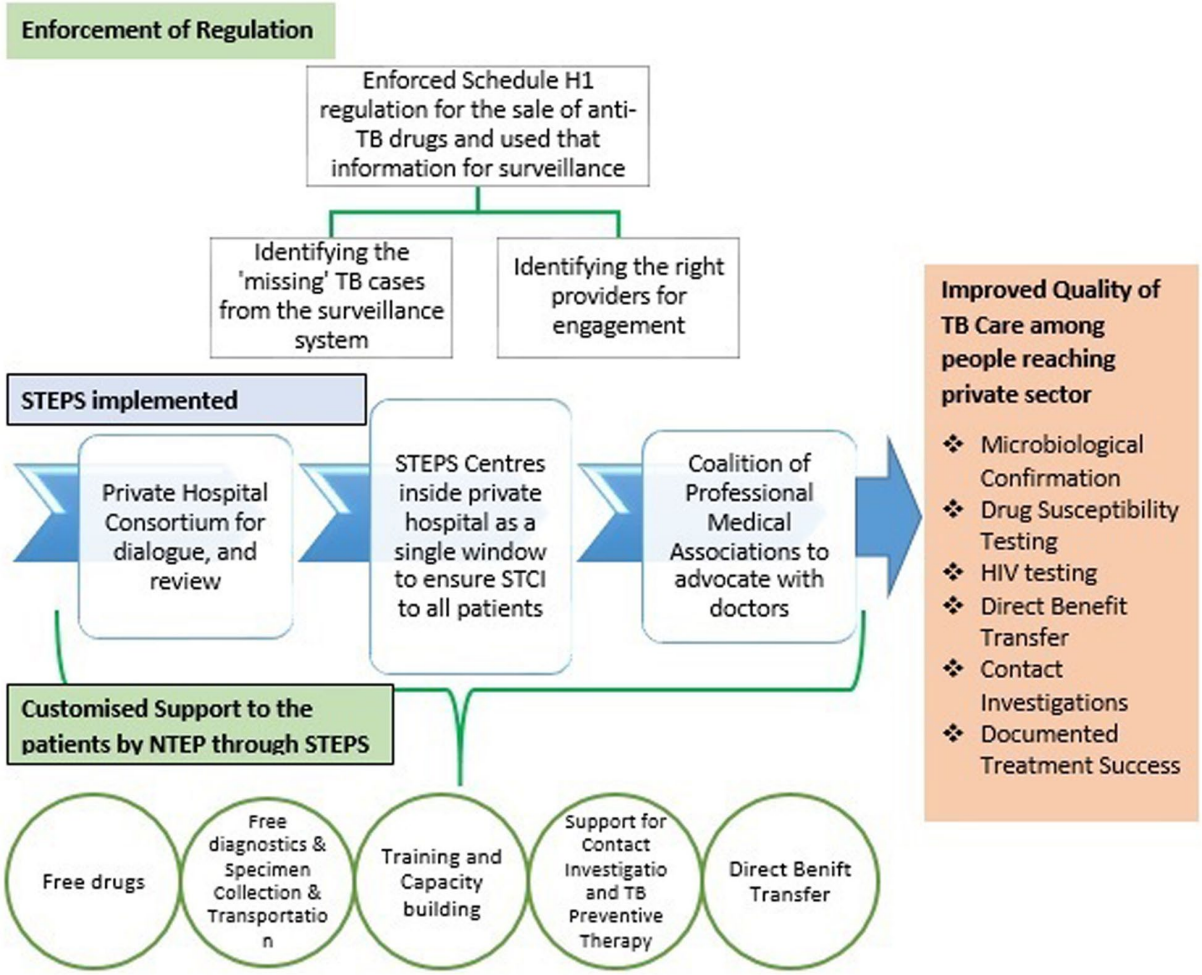
Schematic representation of major initiatives by Kerala to improve the quality of TB care among people reaching the private sector

Since 2016, the Government of Kerala has enforced Schedule H1 implementation for anti-TB drugs as a joint venture by the drugs control department and state TB Elimination program, with monitoring from the top administrative level. From 2018 onwards, the state program managers of NTEP have modified it into a  Schedule H1 surveillance system [28]. The process of schedule H1 surveillance and its benefits were documented through in-depth interviews of the drugs control department enforcement officers, chemist shop owners, private sector doctors, leaders of professional medical associations, and program managers and key staff of the TB Elimination Program in Kerala [28]. The TB Elimination Program of Kerala used the information from the Schedule H1 drug register to identify the missing TB cases from the surveillance system, identify providers who prescribed the anti TB drugs and extended support to them for ensuring STCI to their patients, and provide feedback to providers regarding prescription practices. The estimated number of un-notified TB cases per 100,000 people based on the total sales of rifampicin-containing products in Kerala showed an annual decline of 22% over the last few years, closing the gap in the surveillance system [17]. The major initiatives taken by the state for ensuring quality care to the patients reaching the private sector is represented schematically in Fig. 4.

### Learnings from Kerala for engaging private sector for TB care

1. **Vision of partnership:** Most of the partnership models implemented in the country were mostly business-centred like incentive-based or service-purchase models that were similar to a client-vendor relationship rather than an equal partnership between the public and private sectors. Several models implemented in India that have successfully increased private case notifications were difficult to expand due to lesser emphasis on creating lasting partnerships and huge short-term financial implications [37, 38]. As the intermediary agency who interacts with the private sector withdraws, the model collapses. Kerala state has literally redefined the ‘private sector engagement’ as a partnership for shared health outcomes. The vision of partnership that the state implemented through STEPS is in line with the recent vision shared by World Health Organization which described partnership as a means to “bring together a set of actors for the common goal of improving the health of populations based on mutually agreed roles and principles based on the principles of relative equality between the partners, mutual benefits to the stakeholders, autonomy, accountability and mutual commitment to agreed objectives” [39]. The state has proved that partnership with private sector is possible in the form of a relationship and not mere as contracts or purchase.
2. **Strategic policy direction:** Having a clear policy and strategy will help to avoid confusions among the stakeholders and will enable them to execute better. It should clearly define the goals and objectives of private sector engagement, clarify roles of all stakeholders, describe the institutional arrangements for engagement, outline the feasible strategies and arrangements to monitor performance. Health system decision makers need to know “where they are going” to be able to make efficient use of finite resources. The state has set a clear strategic policy direction with regard to engagement of private sector and trained all program managers regarding the same [29]
3. **Strong governance mechanism:** In Kerala, the state government focused on the governance of the whole health system – both private and public – to ensure access to quality TB care. The government efforts were successful in aligning the private sector with a common goal and making them commit to work to support the agenda. Both the sectors collectively delivered on the realization to ensure access and quality of TB care. Institutional mechanisms for periodic interaction between the public health system and the private sector were established. There was a clear understanding and delineation of the roles based on each stakeholder’s skills and expertise and there was mechanism to ensure accountability of both sectors. There was also a strong regulatory environment with regard to implementation of schedule H1 for anti-TB drugs, at the same time the information through the process has been used for generating data intelligence and ‘nudging’ positive behaviors among the private sector. The governance mechanisms ensured that the actors have the powers to do their jobs and to ensure that others do theirs. The government proactively promoted initiatives like IPAQT where TB tests are made available at affordable prices in the private sector [40]. Through ‘Coalition of Professional Medical Associations’ the government tried to bring in a social regulation too promoting STCI to every citizen.
4. **Understanding the ‘problem’:** Clear understanding of the ‘problem’ that is to be solved through partnership is critical in any successful partnership. The partnership needs to be tailor made to address the identified problems. Similarly understanding the behaviour of stakeholders and involving them from the planning phase are also important before developing any strategy. In Kerala, efforts were put in formally through operations research to identify the problem clearly and document the perceptions and concerns of different stakeholders [32, 33]. STEPS evolved as a solution to address those concerns. Major challenges for ‘partnerships’ identified through various operations research done in the state and how that have been addressed through the STEPS is summarised in Table 2. Kerala experience also reiterates that it is better to avoid over-reliance on pre-designed solutions when it comes to designing partnerships.
5. **Generate data intelligence:** There need to be systematic efforts for collection, compilation and analysis of data to guide the priorities for action. In Kerala, overall data regarding the sales of anti-TB drugs helped in (1) estimating the load of patients being treated in private sector and (2) monitor the trends. Granular information from schedule H1 data helped to (1) identify the potential providers for engagement and (2) identify the cases missed from the official surveillance system [28]. Experiences from the state iterate that pharmacy based surveillance of anti-TB drug sales has immense potential to improve the overall quality of TB care.
6. **Enable stakeholders, foster relations and align structures:** In Kerala, organizational structures were aligned towards the policy objectives that empowered the actors. ‘Private hospital consortium’ and ‘coalition of professional medical associations’ at state and district level enabled actors to work openly, sustainably, and effectively together, with trust. The consortium became the face of the private sector which facilitated dialogues between partners, catalyzed the implementation of policies and ensured that both the sectors are held accountable for their actions for the population. There were also district and sub-district wise WhatsApp groups with STEPS leads of all hospitals and the NTEP staff which facilitated smooth and easy communication. All such efforts to foster relations allowed stakeholders to move beyond simply understanding one another, to being able to work together.
7. **Openness to change**: Important strategic change that contributed to rapid scale-up and acceptance of STEPS were the attempts to gain the trust of hospital management, involvement of nurses for documentation and counselling, efforts for quality control through a coalition of professional medical associations, lack of formal memoranda of understanding, and lack of major financial transactions between partners [30]. Such flexibility and openness to change is crucial while governing mixed health systems.
8. **Patient needs to be at the centre of every partnership:** All public private partnership need to be ‘patient centric’ and their specific health needs and desired health outcomes should be the driving force. STEPS is a patient centric partnership where patient is the central figure in the continuum of care [30, 31]. STEPS tells us that partnerships need to be designed after understanding the needs, preferences and circumstances of patients.
9. **Equipping the public sector with the necessary skills and understanding to effectively engage with private sector:** The state has undertaken a behavior change strategy for the NTEP staff to have a uniform outlook with regard to private sector engagement. Capacity of program managers and peripheral staff were built to deal with private sector in a more efficient way [31].
10. **Incorporating UHC principles into a business model is possible:** STEPS is a model based upon a self-initiated business promotion focusing on the concepts of ‘quality of care’ and ‘customer loyalty’ blended with the social responsibility of the private sector. STEPS centers used ‘after-sales support’ by following up every client, which could also be viewed as a business strategy which typically leads to higher customer satisfaction, brand loyalty, and even word-of-mouth marketing. Cost estimates revealed that implementing STEPS will be of benefit to private hospitals in terms of business returns [31].

**Table 2:** Understanding the barriers for partnerships for TB care and designing solutions

Sl No	Barriers for effective participation of private sector identified through operations research	How STEPS addressed the same?
1	Lack of cohesion and coordination between public and private sector	Private hospital Consortium and Coalition of Medical Professional Associations were formed at state and district level with specific objectives to improve co-ordination.
2	Absence of mutual trust between the public and private sector	Nurtured trust building through early engagement of private sector in designing the process and all the way through in the process including monitoring, review and evaluations.
3	Concerns over patient confidentiality and patient choices	STEPS is a ‘patient centric’ model where the choice remains with the patient. No direct interaction with the people with TB reaching private sector and the staff of public sector.
4	Apprehension of ‘private sector of ‘losing’ patients	No attempt to ‘pull’ the patients from private sector. Rather it fostered the relationship between private hospital and the patients.
5	Lack of consideration for hospital management	Private hospital consortium consisting of private hospital managements was constituted which supports policy decisions and review the performances of STEPS
6	Lack of time for doctors in private sector to document	STEPS Leads and links, who were predominantly nurses, were entrusted with documentation in every private hospital
7	Inability of the TB program to provide timely payments for purchase of services from private	No purchase of services from private sector involved.
8	‘Poor’ recognition felt by the private sector	STEPS is a model owned by the private sector.
9	Bureaucratic hurdles from the public sector in signing contracts for partnership schemes	STEPS is a private hospital led initiative to ensure STCI to their clients and did not involve signing of any contracts .
10	Involving in public health program leads to ‘business loss’ to private sector	STEPS designed as a profitable customer care service model
11	Lack of capacity for public sector staff to understand and deal with private sector	Capacity building and change management undertaken for the staff of public sector for engaging the private sector.
12	Power imbalance between sectors	Conceived as an equal partnership model without any financial transactions
13	Difficult for the NTEP field staff to communicate with private sector	STEPS Leads act as the point of contact for all activities in the concerned hospital. Smooth and rapid communication ensured through WhatsApp groups.
14	Poor public health perspective for private sector	Public health services delivered as customer care services without business loss.
15	Engaging private sector perceived as burden by the public sector staff	Capacity building and change management undertaken for the staff of public sector for engaging the private sector.

## Discussion

The current study documents various approaches and efforts taken by the Kerala TB Elimination program to partner with the‘for profit’ formal private health care delivery providers for ensuring quality TB care. The current study attempts to synthesise the evidences generated from various studies conducted over different time periods and narrates a story which explains the changes in strategies, outcomes and the reasons for the same. Though the results are not meant to be generalized to other settings, it provides lot of insights to strengthen the private sector engagement in the country.

Though, Kerala state was proactive in engaging hospital and laboratories for DOTS (Directly Observed Treatment Short course) through formal memorandum of understanding through various partnership schemes and various efforts for capacity building, a significant chunk of private hospitals could not be engaged until 2018 resulting in sub-optimal quality of care to a significant proportion of people reaching the private sector in terms of not being offered  treatment adherence support, baseline susceptibility testing for Rifampicin, contact investigations and linkages to social welfare schemes. After thorough understanding of the problem and the characteristics of the private sector and in consultation with the stakeholders, the state initiated STEPS which is an equal partnership model where both sectors have been made accountable to ensure uniform standards of TB care to the citizens reaching the private sector. Through enforcing schedule H1 regulations for the sales of anti-TB drugs, the state generated data and established an intelligence system for prioritising actions for partnerships and monitoring the outcomes. The implementation resulted in improvement in the quality of TB care among the people with TB reaching the private sector too.

Through the initiatives, Kerala has literality reframed ‘private sector engagement’ as ‘a partnership where both the sectors come together for the benefit of the society’. STEPS ensured that public resources are not diverted too much; and whatever is diverted through the STEPS is for the patient. In a country like India where most of the patients seek care from the private sector and the program managers are thriving to ensure quality of care in public sector with the limited resources, ‘purchasing’ of services and ‘incentivising’ of the private sector may not be the best way to create sustainable partnerships. Instead, the commitments to offer quality TB services need to be considered as the prime partnership option. Private sector will be more than willing to improve the quality of care to their clients as it improves patient satisfaction and provides good marketing advantage in the competitive healthcare. High out of pocket health expenditure will be the only concern then, which could be addressed to the needy through public provision of services through the private partner, promoting schemes such as IPAQT where TB tests are made available at affordable prices in the private sector or hospitals getting reimbursed for drugs and diagnostics through national health insurance schemes.

Kerala demonstrated that if Governments take a proactive role in engaging the private sector, in an informed and evidence-based way, they can leverage the advantages of the private sector while protecting the public health interest. The case study reiterate that a good governance is essential for ensuring care in a mixed health system. The pursuit of UHC requires Governments to take ownership of healthcare, irrespective of where a person seeks care. WHO Advisory Group on the Governance of the Private Sector for Universal Health Coverage also emphasise a fundamental shift in the Governance behaviours to do business in a new way [39].

Data intelligence is crucial to successful governance. Good governance require data to identify whom to engage, monitor the progress and measure the impact. The state has used data coming out from the drug sales for the surveillance purpose. The state demonstrated that pharmacy based surveillance of anti-TB drug sales has immense potential to help the TB programs in improving the quality of care in private sector.

Success of STEPS also emphasises that successful private sector engagement initiatives need to have a platform for meaningful dialogue. Strengthening platforms, or structures for dialogue, and communication between sectors is important in building trust, and co-development of policies. Medical College Task Force is an example of a structured mechanism where NTEP interacts with all medical colleges [41]. It has state, zonal and national level structures. Similarly, a ‘Private Health Sector Task Force/consortium’ created at district, state, zonal and national level need to be established at the country level.

Kerala state has taken proactive role to partner with private sector after ensuring the quality of care in the public system. The model need to be customised and contextually adapted following the principles and approach and shall not be replicated as such, especially in settings where health systems are weak. It has also to be emphasised that the current experiences with the STEPS model in Kerala are with private hospitals and not with individual practitioners. It is also recommended that Kerala needs to systematically capture the patient’s cost and document the same for more meaningful insights. 

## Conclusion

Kerala state has taken a variety of interventions through STEPS to ensure universal access to TB care for citizens reaching the private sector with documented improvement in the quality of TB care. Kerala demonstrated that if Governments take a proactive role in engaging the private sector, in an informed and evidence-based way, they can leverage the advantages of the private sector while protecting the public health interest. The case study reiterates that a good governance is essential for ensuring care in a mixed health system and such successful governance requires good data intelligence. Kerala reminds us that identifying the correct ‘problems’ are critical for effective design of partnerships and over reliance on pre designed solutions may not work.

## Supplementary Information

**Supplementary Material 1.** Abstract in Malayalam.

## Data Availability

Data are available upon reasonable request. Data collected for the study, including individual de-identified participant data and a data dictionary defining each field in the set, study protocol, informed consent forms will be made available to others for a period of three years from the date of publication of the article, with a signed data access agreement and on due approval from the institutional ethics committee of Amity University, India, on submission of request to the principal investigator explaining the purpose for which the data will be used along with other relevant supporting documents.
