# Supplementary material for: Ensuring universal access to quality care for persons with presumed tuberculosis reaching the private sector: lessons from Kerala
Source: Int J Equity Health. 2024 May 17;23:101. doi: 10.1186/s12939-024-02151-1 (PMC11102222; doi:10.1186/s12939-024-02151-1)
Supplement: Supplementary file 1 — Supplementary Material 1. Abstract in Malayalam. [file 12939_2024_2151_MOESM1_ESM.zip › 2151 Malayalam abstract.pdf]

സംഗ്രഹം

പശ്ചാത്തലം : ഇന്ത്യയിൽ പകുതിയിലേറെ ആളുകൾ ക്ഷയരോഗ ലക്ഷണങ്ങൾക്കു ചികിത്സക്കായി സ്വകാര്യ മേഖലയെ ആശ്രയിക്കുന്നു. കൃത്യമായ രോഗനിർണയ-ചികിത്സാ മാനദണ്ഡങ്ങളുടെ അഭാവത്താലും രോഗബാധിതർ ചികിത്സാ ഇടയ്ക്കു വച്ച് നിർത്താതെ ശരിയായ കാലയളവിൽ മരുന്ന് കഴിച്ചു എന്ന് ഉറപ്പുവരുത്തുവാനുള്ള സംവിധാനങ്ങൾ ഇല്ലാത്തതിനാലും സ്വകാര്യ മേഖലയിൽ നിന്നും ക്ഷയരോഗ ,ചികിത്സ തേടുന്നവർ അത് വിജയകരമായി പൂർത്തിയാകുവാനുള്ള സാധ്യത കുറവായി കണക്കാക്കുന്നു; തദ്ഫലമായി അവരിൽ ഒന്നാം നിര മരുന്നുകളെ പ്രതിരോധിക്കുന്ന ഗുരുതര രോഗത്തിനുള്ള സാധ്യത കൂടുതലായി കണക്കാക്കപ്പെടുന്നു . സ്വകാര്യ മേഖലയിൽ ക്ഷയരോഗ ലക്ഷണങ്ങളുമായി എത്തുന്നവർക്ക് ഉന്നത ഗുണനിലവാരമുള്ള പരിചരണവും അവർക്കും അവരുടെ കുടുംബാംഗങ്ങൾക്കും പൊതുജനാരോഗ്യ നടപടികളും കേരള സംസ്ഥാനം എങ്ങനെ ഉറപ്പാക്കി എന്നത് ശാസ്ത്രയീമായി രേഖപ്പെടുത്തുക എന്നതാണ് ഈ പഠനത്തിന്റെ ലക്ഷ്യം.

പഠനരീതി: അനുബന്ധമായ എല്ലാ രേഖകളും പഠനങ്ങളും ശാസ്ത്രീയമായി പഠിച്ചും ഈ വിഷയത്തിൽ പ്രാഗൽഭ്യമുള്ള അഞ്ച് വിദഗ്ധരെ അഭിമുഖം ചെയ്തും ശേഖരിച്ച വിവരങ്ങൾ ഒരു കേസ് പഠന രീതിയിൽ വിശകലനം നടത്തിയിരിക്കുന്നു .

കണ്ടെത്തലുകൾ: സ്വകാര്യമേഖലയിൽ ക്ഷയരോഗ ലക്ഷണങ്ങളുമായി ചികിത്സ തേടി എത്തുന്ന എല്ലാവർക്കും ഉന്നത ഗുണനിലവാരമുള്ള ചികിത്സയും പരിചരണവും പൊതുജനാരോഗ്യ സേവനങ്ങളും ലഭ്യമാക്കുവാൻ STEPS (സിസ്റ്റം ഫോർ ടിബി എലിമിനേഷൻ ഇൻ പ്രൈവറ്റ് സെക്ടർ) എന്ന പൊതു സ്വകാര്യ പങ്കാളിത്ത പദ്ധതി വഴി കേരളം വ്യവസ്ഥാപിതമായ ശ്രമങ്ങൾ വിജയകരമായി നടത്തിയിട്ടുണ്ട്. കേരളത്തിന്റെ വിജയകരമായ അനുഭവങ്ങളിൽ നിന്നും വെളിവാകുന്ന പാഠങ്ങൾ ഇവ എല്ലാം ആണ് : (i)പൊതുസ്വകാര്യ പങ്കാളിത്തത്തിൻെറ ഗുണഭോക്താവ് സമൂഹം ആയിരിക്കണം , (ii) പൊതുസ്വകാര്യ പങ്കാളിത്തത്തിനു നല്ലൊരു ഭരണഘടന ആവശ്യമാണ് , (iii) പൊതുസ്വകാര്യ പങ്കാളിത്ത പദ്ധതി തയാറാക്കാനും മോണിറ്റർ ചെയ്യുവാനും നല്ല ഡാറ്റ ഇന്റലിജൻസ് ആവശ്യമാണ്, (iv) നിലനിൽക്കുന്ന പ്രശ്നങ്ങളെ ശരിയായി പഠിച്ച ശേഷമായിരിക്കണം പൊതുസ്വകാര്യ പദ്ധതികൾ ആവിഷ്കരിക്കേണ്ടത്, (v) പൊതുസ്വകാര്യ മേഖലകൾ തമ്മിൽ നിരന്തര ആശയവിനിമയത്തിനായുള്ള സംവിധാനങ്ങൾ സജ്ജീകരിക്കേണ്ടതായുണ്ട് .

ഉപസംഹാരം : എല്ലാ പൗരന്മാർക്കും സാർവത്രികമായ ക്ഷയരോഗ നിർണയ ചികിത്സാ സംവിധാനങ്ങൾ ലഭ്യമാക്കുവാൻ പൊതുജനാരോഗ്യ താല്പര്യങ്ങൾ മുൻനിർത്തി സർക്കാർ സംവിധാനങ്ങൾ സ്വകാര്യ മേഖലയെ പങ്കാളി ആക്കിയപ്പോൾ സമൂഹത്തിന് അതിന്റെ ഗുണങ്ങൾ ലഭിച്ചതിന്റെ ഉത്തമ ഉദാഹരണം ആണ് കേരളം നടപ്പിലാക്കിയ STEPS പദ്ധതി.
